# Effect of low-level laser therapy's effectiveness in reducing pain and bone loss around dental implants

**DOI:** 10.6026/973206300214053

**Published:** 2025-11-15

**Authors:** Sajid Ahmed Sanadi, Yogendra Kumar Singh, Mohammed Salah Allahyani, Taha Dafallah, Saurabh Gupta, Abdul Kalam Azad, Savita Singh, Vardharajula Venkat Ramaiah

**Affiliations:** 1 Private Practitioner, Oral and Maxillofacial Surgeon, King Abdullah Medical City, Makkah, Saudi Arabia; 2 Department of Dentistry, Government Medical College, Azamgarh, Uttar Pradesh, India; 3 Private Practitioner, Consultant of Oral and Maxillofacial Surgery, King Abdullah Medical City, Makkah Saudi Arabia; 4 Consultant Oral Maxillofacial Surgeon, Alnoor Specialist Hospital Makkah Mukarama, Kingdom of Saudi Arabia; 5 Private Practitioner, Periodontist and Implantologist, Jammu, Jammu and Kashmir, India; 6 Department of Oral and Maxillofacial Surgery, College of Dentistry, Qassim University, Saudi Arabia; 7 Department of Oral and Maxillofacial Pathology and Microbiology, Private Practitioner, T-265 B, Chirag Delhi, India; 8 Department of Public Health, College of Applied Medical Sciences, Qassim University, Buraydah 51542, PO Box 6666, Saudi Arabia

**Keywords:** Crestal bone loss, implant, laser therapy, pain

## Abstract

The supporting bone structure must be maintained for dental implants to last a long time. Therefore, it is of interest to identify whether post-operative low-level laser therapy (LLLT) of the implant site had any effect on pain alleviation and crestal bone loss surrounding dental implants. Ten individuals in this research who had bilateral tooth loss in the mandibular posterior area received twenty implants, along with LLLT on the test side. Level of crestal bone was assessed radiographically at three and six months and visual analogue scale (VAS) scoring was used to characterise post-operative pain from the start to 6 days. LLLT surrounding the dental implants appreciably decreased post-operative discomfort and loss of crestal bone.

## Background:

Dental implants are preference for replacement lost teeth. Various measures are done to improve the peri-implantitits and pain following implant placement such as low level laser therapy by Light Amplification by Stimulated Emission of Radiation" (LASER) and adjunctive therapies. Implant steadiness is the main clinical measure of osseointegration [1, 2, 3, 4]. For dental implants to last a long time, the supporting bone structure must be maintained. On the contrary to conventional lasers, which are commonly employed to provide thermally damaging and photocoagulation properties, low level lasers are utilised at low power densities to make sure that the target tissue temperature does not rise above the standard (37°C). One innovative therapeutic strategy that has been demonstrated to hasten bone repair is low level laser therapy (LLLT). Numerous studies have demonstrated that LLLT reduces postoperative pain and promotes osteoblast development and proliferation [4]. The laser is a source of non-ionizing radiation that can have non-linear, thermal, or photochemical effects on different tissues, depending on the kind of radiation [5]. The impact of LLLT on early bone loss surrounding dental implants has been the subject of relatively few clinical studies. Therefore, it is of interest to identify low-level laser irradiation on improvement of crestal bone loss and pain sensation surrounding dental implants.

## Materials and Methods:

After obtaining written agreement from participants and approval from the institutional ethics committee, the current study was conducted. After taking inclusion and exclusion criteria into consideration, a skilled researcher conducted this study. Ten participants with bilateral tooth loss in the mandibular posterior region received a total of 20 implants in this split mouth randomised study. The test side was thereafter exposed to low level laser radiation. The coin flip approach was used to identify a group as a test or control. Crestal bone level was examined radiographically at three and six months and visual analogue scale (VAS) scoring was used to measure post-operative pain from the start to 6 days. The statistical assessment of the decrease in pain scores between groups and the loss of crestal bone between groups was done with Mann-Whitney U test and the unpaired t test, correspondingly.

## Results:

No single implants had failed during the study period. Every implant healed without any problems. No significant difficulties were discovered during the course of the trial. The test group's average crestal bone loss at 3 and 6 months was 0.65 and 0.79, whereas the control group's was 1.24 and 1.29. Crestal bone loss varied significantly across the test and control groups (Table 1 - see PDF). The VAS pain levels of the two groups differ appreciably on 2nd and 4th days (Table 2 - see PDF).

## Discussion:

The conservation of the supporting bone surrounding the dental implant identifies its success. Lasers that don't increase body warmth are known as low level or cool lasers. The manner that LLLT enhances tissue repair at the cellular level supports its usage. LLLT treatment of stressed cells resulted in a decrease in oxidative stress, an increase in ATP production and the release of nitric oxide from CcO [4]. This in vitro study investigated the potential otcome of LLLT of the implant site on crestal bone loss and pain alleviation surrounding dental implants. We saw less bone loss and less discomfort in the laser-treated location in comparison to the control group. LLLT surrounding dental implants dramatically reduced crestal bone loss and post-operative discomfort, per Danyasi *et al.*'s findings [4]. Camolesi *et al.* concluded that reducing inflammation and promoting early healing can be achieved well with low laser [6]. Fahmy *et al.* claim that LLLT improves implant stability and has a noticeable impact on bone remodelling [5]. Palled *et al.* concluded that low-level laser treatment may help to heal hard and soft tissues around implants [7]. It was the opposite of what we found. Photobiomodulation approaches can speed up the early phases of osseointegration [8]. Makker *et al.* found considerable decrease in pain score after laser irradication at test side compared to control group. Low level laser helps in repair of tissue and in reducing inflammation and pain [9]. Since photochemical processes, not heat, are responsible for LLLT's therapeutic effects, it is regarded as a nonthermal treatment method [10]. A promising noninvasive therapeutic approach that promotes osteogenesis and speeds up bone regeneration is laser treatment. This is ascribed to its capacity to stimulate osteoblast development and cell proliferation. In overprepared implant sockets, bone growth was considerably enhanced by laser irradiation of the peri-implant bone [11]. Shenoy *et al.* came to the conclusion that LLLT has the potential to improve the stability and osseointegration of dental implants [12]. According to Deljavan *et al.* LLLT can successfully lessen bone resorption when fresh-socket implants are placed [13]. Jaiswal suggested used of digital impression for implant placement [14]. Dental implants are commonly used to replace missing teeth [15, 16, 17, 18, 19, 20- 21]. It was suggested that, Low-level laser therapy appreciably improves periodontal health [22]. A necessary precondition for the long-term success of dental implants is osseointegration. Successful osseointegration has been found to depend on stable blood clot formation and appropriate epithelial tissue healing [15]. More research is needed to validate the results.

## Conclusion:

We may conclude that low intensity irradiation around dental implants appreciably decreased post-operative discomfort and crestal bone loss.
